# Automated Behavioral Analysis of Schizophrenia-like Phenotypes in Repeated MK-801-Treated Mice Using IntelliCage

**DOI:** 10.3390/ijms26115184

**Published:** 2025-05-28

**Authors:** Hisayoshi Kubota, Xinjian Zhang, Masoumeh Khalili, Xinzhu Zhou, Yu Wen, Taku Nagai

**Affiliations:** 1 Division of Behavioral Neuropharmacology, International Center for Brain Science (ICBS), Fujita Health University, Toyoake 470-1192, Aichi, Japan; hisayoshi.kubota@fujita-hu.ac.jp (H.K.);; 2 Fujita Mind-Brain Research & Innovation Center for Drug Generation (Fujita Mind-BRIDGe), Fujita Health University, Toyoake 470-1192, Aichi, Japan

**Keywords:** cognitive flexibility, competitive behavior, IntelliCage, MK-801, schizophrenia

## Abstract

Schizophrenia is a psychiatric disorder characterized by positive, negative, and cognitive symptoms. MK-801, an N-methyl-D-aspartate receptor antagonist, has been used to induce schizophrenia-like behaviors in animal models. Here, we employed IntelliCage, an automated system used for tracking behavior, to assess schizophrenia-like behaviors in MK-801-treated mice under semi-naturalistic conditions. Mice that had been treated with MK-801 for 2 weeks were analyzed for locomotion, emotional, and cognitive functions. Repeated MK-801-treated mice exhibited transient hyperactivity in a novel environment, without significant changes in overall circadian activity. Sucrose preference remained intact, suggesting preserved reward sensitivity. However, less time spent in the corner during the early phase of the competition test indicated reduced competitive behavior for limited water rewards. In the behavioral flexibility test, repeated MK-801-treated mice showed impaired reversal learning, suggesting reduced cognitive flexibility, although the acquisition of initial place discrimination was comparable to that observed in control mice. These behavioral impairments parallel core symptoms of schizophrenia, particularly in the social and cognitive domains. Our findings demonstrate the utility of IntelliCage in detecting behavioral phenotypes over prolonged periods in group-housed settings. This study provides an ecologically valid platform for assessing schizophrenia-like behaviors and may facilitate the development of translationally relevant therapeutic interventions.

## 1. Introduction

Schizophrenia is a severe psychiatric disorder characterized by positive symptoms (e.g., hallucinations and delusions), negative symptoms (e.g., social withdrawal and anhedonia), and cognitive deficits, with an estimated lifetime prevalence of approximately 1% [1]. Animal models play a crucial role in elucidating the underlying neurobiological mechanisms and developing potential therapeutic interventions. Blockade of the N-methyl-D-aspartate (NMDA) receptors has been reported to induce schizophrenia-like symptoms in humans, thereby supporting the glutamate hypothesis, which implicates glutamatergic hypofunction in the pathophysiology of schizophrenia [2]. In line with this hypothesis, a non-competitive NMDA receptor antagonist, namely MK-801, has been widely used to induce schizophrenia-like behaviors in rodents [2]. MK-801 administration in rodents induces a range of schizophrenia-like behaviors, including hyperlocomotion, social deficits, and cognitive impairments [3,4]. Additionally, it has been reported to induce long-lasting behavioral deficits even after drug withdrawal [5], making it suitable for studying schizophrenia in pharmacological animal models. While MK-801-treated mice are a well-established model, their behavioral changes under semi-naturalistic conditions have not been systematically characterized.

Conventional assessments of schizophrenia model animals predominantly depend on behavioral tests involving human handling, such as the locomotor activity test, novel object recognition test, and prepulse inhibition test [3,6,7]. Although these methods provide valuable insights, they may fail to capture spontaneous behaviors in a naturalistic setting, which is essential for a comprehensive understanding of schizophrenia-related impairments. To address this limitation, automated home cage monitoring systems, such as the IntelliCage, have been developed for continuous, unbiased behavioral tracking under semi-naturalistic conditions [8]. In this system, each mouse is implanted with a unique radio frequency identification (RFID) microchip, which allows for individual tracking of behaviors such as activity levels, nose-poke responses, and drinking behavior. The IntelliCage enables the automated assessment of a wide range of emotional and cognitive functions, including competitive behavior, spatial learning, and reversal learning in group-housed mice over extended periods. This approach offers several advantages: mice can be studied without the stress associated with human handling, experiments are not constrained by time or the presence of researchers, and the methodology enhances the reproducibility of behavioral phenotyping across laboratories worldwide.

In this study, we employed the IntelliCage system to evaluate behavioral alterations in repeated MK-801-treated mice, focusing on aspects of exploratory behavior, competitive behavior, and cognitive flexibility under group-housed conditions. Using this automated system, we aimed to provide a more ecologically valid assessment of schizophrenia-like behaviors, potentially uncovering novel behavioral phenotypes that are not easily detectable using conventional methodologies. This study may contribute to refining behavioral models of schizophrenia and improving translational research in psychiatric disorders.

## 2. Results

### 2.1. Exploratory Behavior and Circadian Activity in Repeated MK-801-Treated Mice

**Figure 1:**
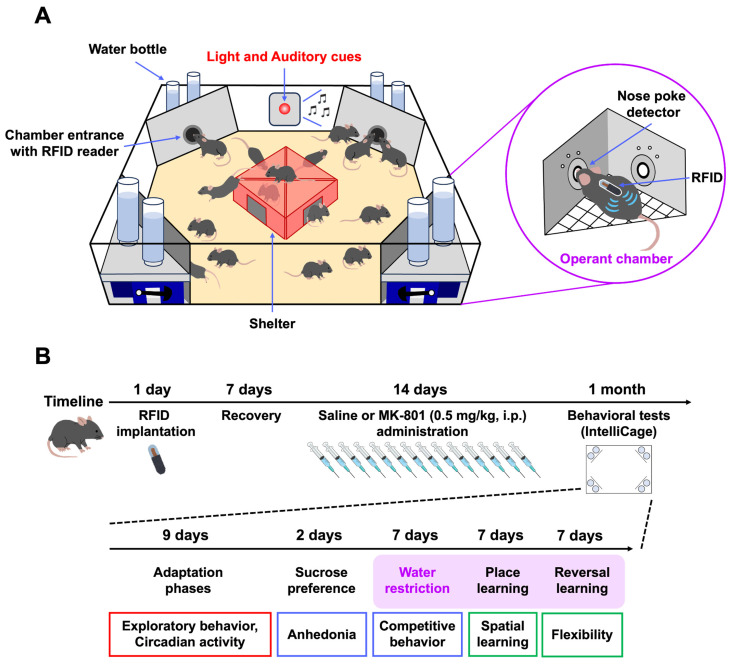
A series of behavioral tests using IntelliCage. (**A**) Schematic representation of the IntelliCage apparatus. RFID-tagged mice were group-housed, and their behavioral responses, namely corner visits, nose pokes, and licks, were automatically monitored. When a mouse visited a corner to obtain a water reward, the RFID reader detected and recorded the mouse’s unique ID number. Test sessions were cued using a red LED on the IntelliCage wall, which emitted both light and sound. (**B**) Scheme of the behavioral tests. Mice were administered MK-801 (0.5 mg/kg, i.p.) for 2 weeks to induce the schizophrenia model and were subsequently housed in the IntelliCage. The behavioral tests were performed sequentially as follows: (1) adaptation phase, (2) sucrose preference test, (3) competition test, and (4) behavioral flexibility test. The purple background indicates the water restriction period.

**Figure 2:**
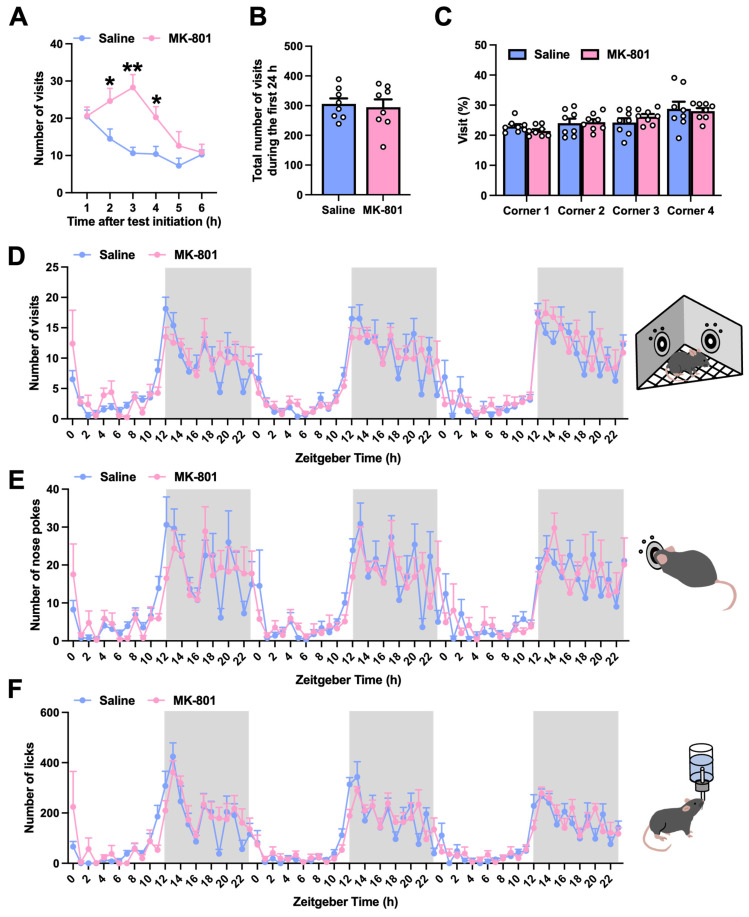
Exploratory behavior and circadian activity in repeated MK-801-treated mice. During the 9-day adaptation phase, exploratory behavior and circadian activity were evaluated by continuously recording the number of visits, nose pokes, and licks, as well as the percentage of visits to each corner. (**A**) Number of visits during the first 6 h. (**B**) Total number of visits and (**C**) percentage of visits to each corner during the first 24 h. (**D**–**F**) Circadian activity was recorded over the last 3 days, based on Zeitgeber times (h, 0 = lights on). Mice were maintained on a consistent 12 h light/dark cycle, with white and gray backgrounds indicating periods of light and darkness, respectively. (**D**) Number of visits, (**E**) nose pokes, and (**F**) licks were plotted every 1 h. Each column represents the mean ± SEM (*n* = 8). * *p* < 0.05, ** *p* < 0.01 versus MK-801.

In the present study, mice were treated with MK-801 (0.5 mg/kg) for 2 weeks. Following the repeated MK-801 treatment period, their behavioral changes under semi-naturalistic conditions were assessed using the IntelliCage system (Figure 1A,B). First, exploratory behavior and circadian activity were analyzed. Repeated MK-801-treated mice exhibited an increased number of corner visits, which peaked 3 h after the test initiation (Figure 2A). However, no significant changes were observed over the entire 24 h period (Figure 2B). No pre-existing biases were found in the percentage of visits to each corner over 24 h between the groups (Figure 2C). Regarding circadian activity, we analyzed the number of visits, nose pokes, and licks over 72 h. A clear distinction between the active (dark) and inactive (light) periods was exhibited by both the repeated saline- and MK-801-treated mice, with no significant differences between the two groups (Figure 2D–F). These results suggest that repeated MK-801-treated mice exhibited transient hyperactivity in a novel environment, without affecting overall circadian activity patterns.

### 2.2. Sucrose Preference and Competitive Behavior in Repeated MK-801-Treated Mice

**Figure 3:**
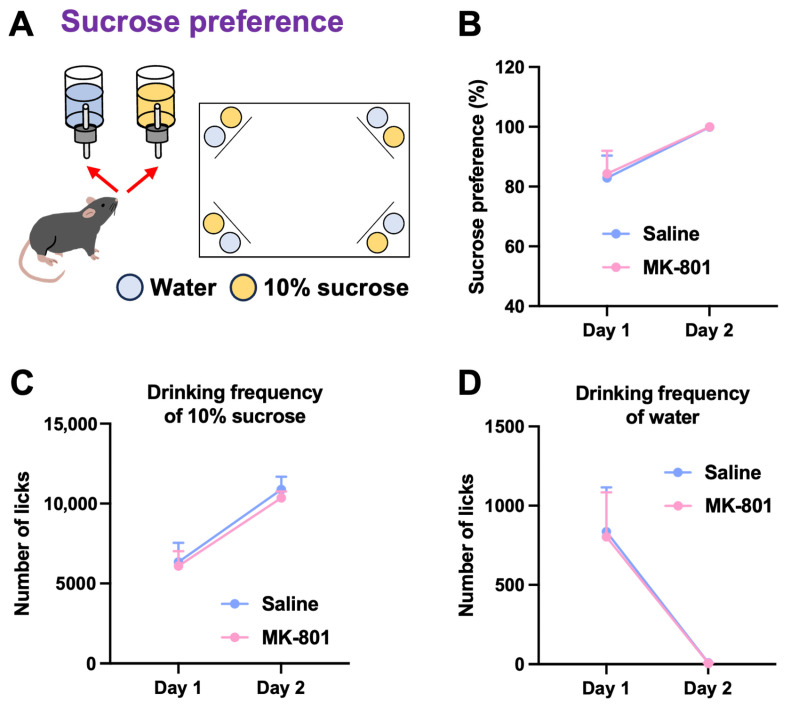
Sucrose preference in repeated MK-801-treated mice. (**A**) Schematic representation of the sucrose preference test. In each corner, one of the bottles was filled with water and the other with a 10% sucrose solution. Sucrose preference was calculated as the percentage of licks on the sucrose bottle relative to the total number of licks. (**B**) Sucrose preference, (**C**) number of licks on the sucrose bottles, and (**D**) number of licks on the water bottles were recorded for 2 days. Each column represents the mean ± SEM (*n* = 6).

**Figure 4:**
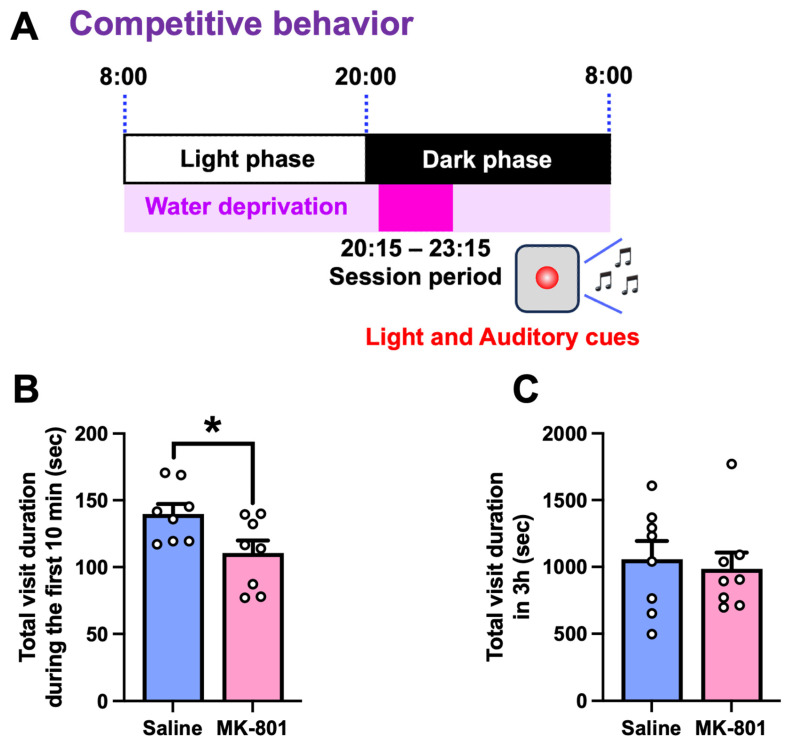
Competitive behavior in repeated MK-801-treated mice. (**A**) Timeline of the competition test. Mice were water-restricted at all times, except during the 3 h test sessions (20:15 to 23:15) for 7 days. A red LED on the cage wall provided light and auditory cues during the test sessions. On day 7, the time spent visiting each corner during (**B**) the first 10 min and (**C**) the full 3 h was recorded. Each column represents the mean ± SEM *(n* = 8). * *p* < 0.05 versus MK-801.

To evaluate emotional functions, repeated MK-801-treated mice were subjected to the sucrose preference test (Figure 3A) and the competition test (Figure 4A). Repeated MK-801-treated mice showed a higher sucrose preference on day 2 than on day 1, with no significant differences between the two groups (Figure 3B). This was supported by an increased number of licks on the sucrose bottles (Figure 3C) and a decreased number of licks on the water bottles (Figure 3D) on day 2. These results suggest that the sucrose preference of repeated MK-801-treated mice was comparable to that of saline-treated mice, indicating no change in reward-related behavior.

In the competition test, mice were subjected to water restriction throughout the experiment, except during the 3 h test sessions (20:15–23:15), when water was available as a reward (Figure 4A). During the first 10 min of the test, repeated MK-801-treated mice exhibited a significantly shorter corner visit duration than saline-treated mice (Figure 4B). However, the total visit duration over the entire 3 h session did not differ between the two groups, indicating that repeated MK-801 treatment has no effect on the motivation for water rewards (Figure 4C). These results suggest that MK-801-treated mice exhibited reduced competitive behavior in a group-housed setting.

### 2.3. Cognitive Flexibility in Repeated MK-801-Treated Mice

**Figure 5:**
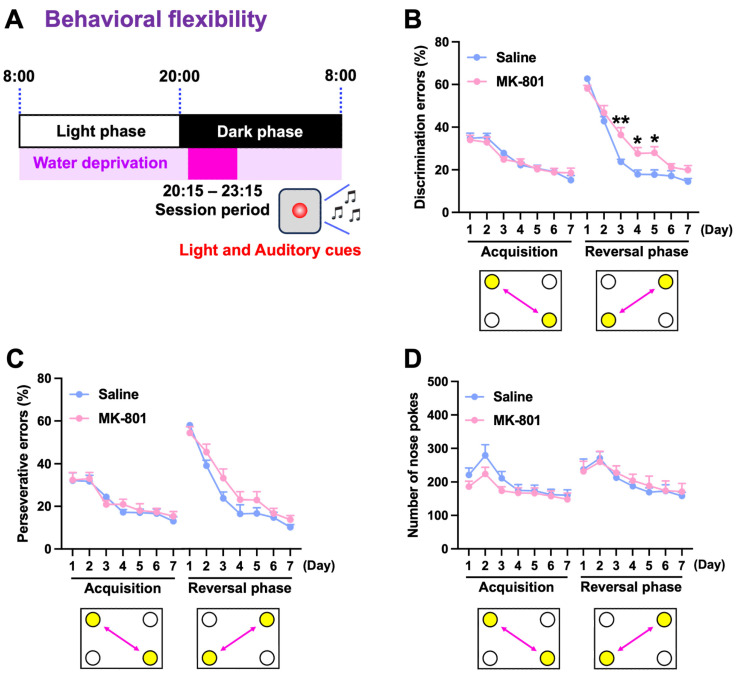
Cognitive flexibility in repeated MK-801-treated mice. (**A**) Timeline of the behavioral flexibility test comprising acquisition and reversal phases, each lasting 7 days. Mice were water-restricted at all times, except during the 3 h test sessions (20:15 to 23:15). A red LED on the cage wall provided light and auditory cues during the test sessions. The percentages of (**B**) discrimination errors and (**C**) perseverative errors, as well as (**D**) the number of nose pokes, within the first 100 visits of each session were recorded. Each column represents the mean ± SEM (*n* = 8). * *p* < 0.05, ** *p* < 0.01 versus MK-801.

To evaluate cognitive functions, repeated MK-801-treated mice were subjected to the behavioral flexibility test, which consisted of both an acquisition phase and a reversal phase. Mice were allowed to drink water during the 3 h test sessions (Figure 5A). During the acquisition phase, mice had to discriminate and shuttle between diagonally positioned rewarded and non-rewarded corners. MK-801-treated mice demonstrated a comparable ability to that of saline-treated mice, as evidenced by a reduction in the percentage of discrimination errors across seven consecutive daily sessions (Figure 5B). During the reversal phase, the rewarded and non-rewarded corners were switched to assess cognitive flexibility. Saline-treated mice showed the capacity to adapt and effectively discriminate between the novel corners that were either rewarded or not rewarded, whereas the repeated MK-801-treated mice exhibited considerable delays in acquiring novel discrimination, suggesting an impairment in cognitive flexibility (Figure 5B). There were no differences in the percentage of perseverative errors or the number of nose pokes between the two groups throughout the sessions (Figure 5C,D). These results suggest that repeated MK-801-treated mice showed intact place discrimination but had difficulty adapting to new rules, indicating the impairment of cognitive flexibility.

## 3. Discussion

In this study, we evaluated schizophrenia-like behaviors in mice treated with repeated MK-801, an NMDA receptor antagonist, using the automated IntelliCage system. Unlike conventional behavioral testing methods, IntelliCage enables long-term and non-invasive monitoring of group-housed mice in a semi-natural environment, making it a promising approach for detecting behavioral phenotypes that cannot be captured through short-term task-based assays. Notably, repeated MK-801-treated mice exhibited schizophrenia-like behaviors, including increased exploratory behavior in a novel environment, reduced competitive behavior, and impaired cognitive flexibility.

Following exposure to a novel environment, repeated MK-801-treated mice exhibited a significant increase in the number of corner visits, which peaked during the first 3 h, suggesting a transient hyperactivity. In contrast, no significant differences were observed in overall exploratory behavior or circadian activity over 24 h. Repeated MK-801-treated mice may be slightly sensitive and excitable to novel environments, resulting in transient hyperactivity. Increased sensitivity to novelty and abnormal attentional responses have also been reported in patients with schizophrenia [9,10]. This phenomenon is explained by the concept of “aberrant salience”, in which hyperdopaminergic states result in the excessive attribution of significance to irrelevant stimuli [11,12]. Accordingly, the transient hyperactivity in a novel environment in repeated MK-801-treated mice may reflect the pathological sensitivity to novelty observed in schizophrenia.

In the sucrose preference test, repeated MK-801-treated mice retained normal reward sensitivity. We used a relatively high concentration of sucrose (10%) as a reward stimulus, which may have served as a powerful motivator for mice, thereby obscuring the potential differences between groups. Future studies should consider using lower concentrations of sucrose solution (e.g., 1%) to increase the sensitivity of the assay. To further evaluate the motivational aspects of reward-seeking behavior, it may be helpful to implement a progressive ratio task in which the required number of nose-pokes to obtain sucrose gradually increases [13]. This approach would allow for a quantitative assessment of the effort that mice are willing to exert to obtain a reward, thereby providing insight into their level of motivation. In contrast, in the competition test, repeated MK-801-treated mice showed a reduced visit duration during the first 10 min of the test session, indicating decreased competitive behavior toward limited resources (water access). This behavioral deficit is indicative of an impairment in social motivation or withdrawal, which may correspond to the social dysfunction commonly observed in schizophrenia [1]. Notably, reduced hierarchical status has been reported in patients with schizophrenia, reflecting their difficulty in asserting themselves or navigating competitive social interactions [14,15]. Our findings suggest that the repeated MK-801-treated mice exhibit certain aspects of these social behavioral abnormalities.

In the behavioral flexibility test, repeated MK-801-treated mice acquired initial spatial discrimination, similar to control mice, but exhibited delayed adaptation when the reward location was reversed, unlike control mice. This behavioral inflexibility may be associated with the inability to update previously learned associations and suppress ineffective strategies. These processes are critically dependent on the integrity of the prefrontal cortex and its interactions with the hippocampus and striatum [16,17]. Such deficits are highly relevant to schizophrenia, where impaired cognitive flexibility is a core feature, often manifesting as perseverative behavior, rigid thinking, and an inability to adjust to changing environmental demands [18,19]. Therefore, the reversal learning impairment observed in repeated MK-801-treated mice may be due to dysfunction in cortico-subcortical circuits involved in cognitive flexibility. This supports the utility of this model for investigating the neural substrates of cognitive impairments in schizophrenia.

However, several limitations must be acknowledged in this study. First, although we employed a repeated MK-801 administration model based on the glutamate hypothesis of schizophrenia, the multifaceted pathophysiology of the disorder was not fully replicated. Future studies should include comparative analyses using alternative models based on neurodevelopmental hypotheses, such as the poly I:C model or maternal separation paradigm [7,20], which focus on pre- and perinatal neurodevelopmental processes. In addition, we did not assess acute pharmacological responsiveness to psychostimulants (e.g., MK-801 and amphetamine), which can be used to evaluate positive symptoms such as hyperactivity [21]. Other symptom domains, including emotional flattening and attentional deficits, were not comprehensively addressed. The development and application of behavioral paradigms capable of capturing the full spectrum of negative and cognitive symptoms are critical. To examine the predictive validity of the present model, it is also necessary to evaluate whether existing antipsychotic drugs (e.g., risperidone and clozapine) can ameliorate the observed behavioral phenotypes. Furthermore, we did not include measurements of body weight throughout the experimental period, which could have provided important insights into the physiological effects of repeated MK-801 administration. Monitoring such physiological parameters is essential to comprehensively evaluate the systemic impact of the treatment and ensure animal welfare.

Despite the need for additional research, this study comprehensively evaluated schizophrenia-like behaviors in repeated MK-801-treated mice using the IntelliCage. The experimental platform established in this study provides a promising foundation for future investigations into the molecular and circuit-level mechanisms underlying behavioral deficits in MK-801-treated mice under semi-naturalistic conditions. Our findings highlight the utility of group-housed repeated MK-801-treated mice for investigating schizophrenia-like behaviors, further promoting the advancement of translational research in psychiatric disorders.

## 4. Materials and Methods

### 4.1. Mice

Male C57BL/6N mice were obtained from Japan SLC (Shizuoka, Japan). The mice were housed in a specific pathogen-free environment within our animal facility prior to use and were maintained in a regulated environment (23 ± 3 °C and 50 ± 10% humidity) with a 12 h light/dark cycle (lights on at 8:00 a.m., off at 8:00 p.m.). The mice were provided food (MF, Oriental Yeast Co., Ltd., Tokyo, Japan) and tap water ad libitum. All mice experiments were approved (number: APU22026-MD9) and performed in accordance with the guidelines for the care and use of laboratory animals established by the Animal Experiments Committee of Fujita Health University.

### 4.2. Drug Administration

MK-801 hydrogen maleate (Sigma-Aldrich, St. Louis, MO, USA, #M107) was dissolved in 0.9% saline. MK-801 (0.5 mg/kg) was intraperitoneally (i.p.) administered to mice for 2 weeks [5].

### 4.3. IntelliCage

The IntelliCage system (TSE Systems GmbH, Bad Homburg, Germany) is a computerized, fully automated apparatus designed for studying spontaneous emotional and cognitive behaviors in group-housed RFID-tagged mice within a large home cage (Figure 1A). This system consists of a spacious plastic cage (55 cm × 37.5 cm × 20.5 cm) featuring four triangular operant chambers (referred to as corners, 15 cm × 15 cm × 21 cm). It also contains RFID readers, infrared sensors, and lickometers, enabling simultaneous monitoring of up to 16 RFID-tagged mice within a single IntelliCage unit. The design ensures that only one mouse can enter a corner, due to the restricted size of the tunnel and corner. Inside the corner, mice interact with two nose-poke holes equipped with infrared detectors that register beam-break responses. A nose poke triggers the opening of a motorized gate, providing access to water bottle nipples. The IntelliCage system automatically records behavioral events (i.e., corner visits, nose pokes, licks), the mouse ID, and the corner ID, using the RFID readers, infrared sensors, and lickometers.

### 4.4. Behavioral Tests

At 8 weeks of age, mice were anesthetized by i.p. administration of a mixture containing medetomidine (0.75 mg/kg, Nippon Zenyaku Kogyo, Fukushima, Japan), midazolam (4 mg/kg, Sandoz, Basel, Switzerland), and butorphanol (5 mg/kg, Meiji Seika Pharma, Tokyo, Japan), and then implanted with a subcutaneous RFID, followed by a week of recovery (Figure 1B). Afterward, the mice were administered MK-801 (0.5 mg/kg, i.p.) for 2 weeks to induce the schizophrenia model and were subsequently housed in the IntelliCage. The behavioral tests were conducted in the following order: (1) adaptation phase, (2) sucrose preference test, (3) competition test, and (4) behavioral flexibility test (Figure 1B).

#### 4.4.1. Adaptation Phase

For the first 3 days, all doors were open to allow free access to the water bottles and allow the mice to acclimate to their new environment. Next, the mice were allowed to open the doors with a nose poke, which provided access to water for 6 days. Each door could be opened only once per corner visit and closed after 5 s. To assess exploratory behavior and circadian activity, the number of visits, nose pokes, and licks, as well as the percentage of visits to each corner, were continuously monitored during this phase. Circadian activity was specifically analyzed during the last 3 days.

#### 4.4.2. Sucrose Preference Test

The sucrose preference test was performed as previously described [13,22] with minor modifications. In each corner, one of the bottles was filled with water and the other with a 10% sucrose solution. Mice were free to choose between the two bottles via a nose poke throughout the day. The number of licks on the sucrose and water bottles was recorded for 2 days. Sucrose preference was calculated as the percentage of licks on the sucrose solution relative to the total number of licks. Mice that never licked the sucrose bottle during the study were excluded from the analysis because they may not have noticed the sucrose bottle.

#### 4.4.3. Competition Test

The competition test was performed as previously described [23] with minor modifications. Mice were habituated to water restriction for 7 days. During the task, they were deprived of water for 21 h per day and allowed to drink water for 5 s per corner visit between 20:15 and 23:15. A red LED that emitted both light and sound was mounted on the side wall of the cage, serving as a visual and auditory cue for 3 h. On day 7, to evaluate competitive behavior for limited water rewards, the time spent visiting each corner during the first 10 min and over 3 h was recorded according to previous report [23].

#### 4.4.4. Behavioral Flexibility Test

The behavioral flexibility test was performed as previously described [23] with minor modifications. This test consisted of an acquisition phase focused on place learning, followed by a reversal learning phase. During the 7-day acquisition phase, mice were rewarded with 5 s of water access whenever they visited specifically assigned corners during the daily 3 h session (20:15–23:15). To obtain rewards, the mice were required to alternate visits between two diagonally positioned corners, learning the locations of these two distant reward sites and shuttling between them. The assignment of reward corners was counterbalanced between groups. Following the acquisition phase, mice underwent a 7-day reversal learning phase, in which the spatial pattern of the rewarded corners was switched along the diagonal axis. The mice had to adjust their shuttling behavior to the newly assigned diagonal reward locations. Learning performance was assessed by counting the number of discrimination errors made within the first 100 visits of each session. Visits to never-rewarded corners were considered discrimination errors, while the number of nose pokes in these never-rewarded corners was regarded as perseverative errors. The total number of nose pokes was used as an indicator of motivation.

### 4.5. Statistical Analysis

All data are expressed as the mean ± standard error of the mean (SEM). Statistical analyses were performed using GraphPad Prism 10 (GraphPad Software Inc., San Diego, CA, USA). Two-way ANOVA followed by Tukey’s post hoc test was employed for statistical analyses with multiple group comparisons. Differences between groups were analyzed using the Mann–Whitney U test (nonparametric data). Detailed statistical methods are described in Table S1.

## 5. Conclusions

In the present study, we demonstrated that repeated MK-801-treated mice exhibited schizophrenia-like behaviors, including transient hyperactivity in a novel environment, reduced competitive behavior, and impaired cognitive flexibility. Using the IntelliCage system allowed for long-term and non-invasive assessment in a group-housed setting. The model successfully captured key aspects of social and cognitive dysfunction. Behavioral analysis under semi-naturalistic conditions using the IntelliCage system is expected to refine disease modeling and improve translational research in psychiatric disorders.

## Data Availability

The datasets are available from the corresponding author upon reasonable request.

## Supplementary Materials

The following supporting information can be downloaded at: https://www.mdpi.com/article/10.3390/ijms26115184/s1.

## Abbreviations

The following abbreviations are used in this manuscript:

NMDA	N-methyl-D-aspartate
RFID	radio frequency identification
