# Supplementary material for: Automated Behavioral Analysis of Schizophrenia-like Phenotypes in Repeated MK-801-Treated Mice Using IntelliCage
Source: Int J Mol Sci. 2025 May 28;26(11):5184. doi: 10.3390/ijms26115184 (PMC12155132; doi:10.3390/ijms26115184)
Supplement: Supplementary file 1 [file ijms-26-05184-s001.zip › ijms-3658054-supplementary.pdf]

International Journal of *Molecular Sciences*

MDPI

Article

Automated Behavioral Analysis of Schizophrenia-like Phenotypes in Repeated MK-801-Treated Mice Using IntelliCage

Hisayoshi Kubota[1,2], Xinjian Zhang[1,2], Masoumeh Khalili[1], Xinzhu Zhou[1], Yu Wen[1], Taku Nagai[1,2,*]

Table S1. Summary of statistical analyses.

Figure	Number	Statistical analysis	Degrees of freedom and F/U/*p* value
Figure 2A	$n = 8$	Two-way repeated measures ANOVA followed by Tukey's multiple comparison test	F_{Time} (3.5, 49.3) = 11.62, $p < 0.01$, F_{MK-801} (1, 14) = 7.91, $p < 0.05$, $F_{Time \times MK-801}$ (5, 70) = 5.73, $p < 0.01$
Figure 2B	$n = 8$	Mann-Whitney U-test	U = 30, $p = 0.88$
Figure 2C	$n = 8$	Two-way repeated measures ANOVA followed by Tukey's multiple comparison test	F_{Corner} (1.8, 25.5) = 6.03, $p < 0.01$, F_{MK-801} (1, 14) = 0.20, $p = 0.66$, $F_{Corner \times MK-801}$ (3, 42) = 0.51, $p = 0.68$
Figure 2D	$n = 8$	Two-way repeated measures ANOVA followed by Tukey's multiple comparison test	F_{Time} (8.9, 125) = 16.01, $p < 0.01$, F_{MK-801} (1, 14) = 0.14, $p = 0.72$, $F_{Time \times MK-801}$ (71, 994) = 1.24, $p = 0.09$
Figure 2E	$n = 8$	Two-way repeated measures ANOVA followed by Tukey's multiple comparison test	F_{Time} (9.4, 131.9) = 10.02, $p < 0.01$, F_{MK-801} (1, 14) = 0.01, $p = 0.91$, $F_{Time \times MK-801}$ (71, 994) = 1.29, $p = 0.06$
Figure 2F	$n = 8$	Two-way repeated measures ANOVA followed by Tukey's multiple comparison test	F_{Time} (7.6, 106.1) = 15.38, $p < 0.01$, F_{MK-801} (1, 14) = 0.32, $p = 0.58$, $F_{Time \times MK-801}$ (71, 994) = 1.44, $p < 0.05$
Figure 3B	$n = 6$	Two-way repeated measures ANOVA followed by Tukey's multiple comparison test	F_{Day} (1, 10) = 9.37, $p < 0.05$, F_{MK-801} (1, 10) = 0.02, $p = 0.90$, $F_{Time \times MK-801}$ (1, 10) = 0.02, $p = 0.90$
Figure 3C	$n = 6$	Two-way repeated measures ANOVA followed by Tukey's multiple comparison test	F_{Day} (1, 10) = 61.96, $p < 0.01$, F_{MK-801} (1, 10) = 0.12, $p = 0.73$, $F_{Time \times MK-801}$ (1, 10) = 0.06, $p = 0.81$
Figure 3D	$n = 6$	Two-way repeated measures ANOVA followed by Tukey's multiple comparison test	F_{Day} (1, 10) = 17.05, $p < 0.01$, F_{MK-801} (1, 10) = 0.01, $p = 0.93$, $F_{Time \times MK-801}$ (1, 10) = 0.01, $p = 0.94$
Figure 4B	$n = 8$	Mann-Whitney U-test	U = 11, $p < 0.05$
Figure 4C	$n = 8$	Mann-Whitney U-test	U = 28, $p = 0.72$
Figure 5B (Acquisition)	$n = 8$	Two-way repeated measures ANOVA followed by Tukey's multiple comparison test	F_{Day} (3.9, 54.3) = 44.48, $p < 0.01$, F_{MK-801} (1, 14) = 0.03, $p = 0.87$, $F_{Time \times MK-801}$ (6, 84) = 1.01, $p = 0.43$
Figure 5B (Reversal)	$n = 8$	Two-way repeated measures ANOVA followed by Tukey's multiple comparison test	F_{Day} (2.9, 40.6) = 149.7, $p < 0.01$, F_{MK-801} (1, 14) = 7.61, $p < 0.05$, $F_{Time \times MK-801}$ (6, 84) = 4.84, $p < 0.01$
Figure 5C (Acquisition)	$n = 8$	Two-way repeated measures ANOVA followed by Tukey's multiple comparison test	F_{Day} (3.7, 51.3) = 28.26, $p < 0.01$, F_{MK-801} (1, 14) = 0.12, $p = 0.73$, $F_{Time \times MK-801}$ (6, 84) = 0.65, $p = 0.59$

Figure 5C (Reversal)	$n = 8$	Two-way repeated measures ANOVA followed by Tukey's multiple comparison test	F_{Day} (3.5, 49.4) = 78.40, $p < 0.01$, $F_{MK\text{-}801}$ (1, 14) = 2.56, $p = 0.13$, $F_{Time \times MK\text{-}801}$ (6, 84) = 1.39, $p = 0.23$
Figure 5D (Acquisition)	$n = 8$	Two-way repeated measures ANOVA followed by Tukey's multiple comparison test	F_{Day} (2.8, 39.1) = 18.68, $p < 0.01$, $F_{MK\text{-}801}$ (1, 14) = 1.06, $p = 0.32$, $F_{Time \times MK\text{-}801}$ (6, 84) = 1.62, $p = 0.15$
Figure 5D (Reversal)	$n = 8$	Two-way repeated measures ANOVA followed by Tukey's multiple comparison test	F_{Day} (2.9, 40.1) = 12.47, $p < 0.01$, $F_{MK\text{-}801}$ (1, 14) = 0.07, $p = 0.80$, $F_{Time \times MK\text{-}801}$ (6, 84) = 0.34, $p = 0.91$
